# Contribution of ^**18**^F-Fluoro-ethyl-tyrosine Positron Emission Tomography to Target Volume Delineation in Stereotactic Radiotherapy of Malignant Cranial Base Tumours: First Clinical Experience

**DOI:** 10.1155/2012/412585

**Published:** 2012-10-08

**Authors:** Reinhold Graf, Michail Plotkin, Fonyuy Nyuyki, Peter Wust, Reinhard Wurm, Volker Budach, Winfried Brenner, Daniel Fahdt

**Affiliations:** ^1^Department of Radiation Oncology, Charité Universitätsmedizin Berlin, Augustenburger Platz 1, 13353 Berlin, Germany; ^2^Department of Nuclear Medicine, Charité Universitätsmedizin Berlin, Augustenburger Platz 1, 13353 Berlin, Germany; ^3^Department of Radiation Oncology, Klinikum Frankfurt (Oder), Müllroser Chaussee 7, 15236 Frankfurt, Germany

## Abstract

Increased amino acid uptake has been demonstrated in intracerebral tumours and head and neck carcinomas of squamous cell origin. We investigated the potential impact of using ^18^F-fluoro-ethyl-tyrosine (^18^F-FET)-PET/CT in addition to conventional imaging for gross tumour volume (GTV) delineation in stereotactic radiotherapy of skull base tumours. The study population consisted of 14 consecutive patients with cranial base tumours (10 with squamous cell histology, 4 others). All patients underwent a FET-PET/CT examination in addition to contrast-enhanced CT and 11 patients underwent MRI. All tumours and histologic types showed increased FET uptake. The GTV was defined by all voxels showing hyperintensity in MRI or CT (GTV_MRI/CT_) or enhancement in PET (GTV_PET_), forming a GTV_composite_ that was used for the initial treatment fields. An additional volume of infiltrative growth outside the GTV_MRI/CT_ of about 1.0 ± 2 cm^3^ (5% of the conventional volume) was demonstrated by FET-PET only (GTV_PETplus_) with significant enlargement (>10% of GTV_MRI/CT_) in three patients. From existing data, we found correlation between cellular density and the standardized uptake value (SUV) of FET. We were able to substantially reduce the volume of escalated radiation dose (GTV_boost_) by 11 ± 2 cm^3^ (24%) of the conventional volume.

## 1. Introduction

 It is assumed that the larger part of geometrical uncertainties in fractionated stereotactic radiotherapy (FSRT) is due to delineation errors during the treatment planning procedure [1]. This is especially serious if the errors lead to marginal tumour misses, resulting in a dismal prognosis, or to enlargement of the volume treated, increasing the frequency of severe late effects. Structures of the skull base (SB) with high signal intensity and high contrast-enhancement in magnetic resonance imaging (MRI) make it difficult to differentiate tumour tissue from normal structures [2] and to exactly delineate the target volume. Therefore, although costly, functional imaging is increasingly used for target volume delineation in SB radiotherapy. The diagnostic value of 2-((18)F)-fluoro-2-deoxy-D-glucose positron emission tomography (FDG-PET) for imaging intracranial tumours is hampered by the low imaging contrast between tumourous tissue and that of the normal brain due to the high glucose utilization of both and this may also be true for SB tumours and the neighbouring brain tissue [3]. The newly introduced tracer O-(2-[^18^F] Fluoro-Ethyl)-L-Tyrosine (^18^F-FET) allows a more precise estimation of intracerebral tumour borders than MRI [4]. Pauleit et al. [5] investigated the diagnostic potential of FET-PET in patients with primary squamous cell cancer (SCC) of the head and neck and found that FET-PET has lower sensitivity (75% versus 93%) but a substantially higher specificity (95% versus 79%) for detecting tumours compared with FDG-PET. Grosu et al. [6] found a high specificity for all four brain metastases evaluated for differentiating tumour from treatment related changes. A strong correlation between cellular density and the standardized uptake value (SUV) of FET has been demonstrated by various study groups [7–9]. Amino acid accumulation provides the ability to boost the radiation dose to the subvolume of the most proliferative region within a tumour as opposed to the initial, larger volume [10, 11]. An integrated-boost dose escalation concept, based on a preirradiation FET-PET guided target volume delineation, has already been initiated by Piroth et al. [12].

 Considering the data of Pauleit et al. [5], we hypothesized that FET-PET might be useful for the determination of tumour borders in scull base malignancies and could also contribute to delineation of intracerebral extension. In the present study, we performed FET-PET/CT in a group of 14 patients to assess its potential contribution to the definition of the GTV in malignant cranial base tumours treated with fractionated stereotactic radiotherapy.

## 2. Materials and Methods

 Between February 2006 and May 2009, in the Department of Radiotherapy and Radiooncology of Charité, a group of 14 consecutive patients with histologically proven malignant SB tumours and evidence of SB bone infiltration in conventional imaging underwent MRI (11 patients) and FET-PET/CT (with contrast-enhanced CT) prior to the start of FSRT. Patient characteristics are presented in Table 1. The majority of patients (10/14) had SCC; while, the other four patients had other histologies. Most (11/14) patients were pretreated. The study was performed in accordance with the Declaration of Helsinki and the protocol was approved by the ethics committee of our institution. Written informed consent was obtained from all patients before enrolment into the study.

 PET data were obtained in 3-dimensional mode using a hybrid PET/CT system consisting of a multislice CT and a full-ring PET scanner (Biograph 16, Siemens Medical Solutions, Erlangen, Germany). A protein-low diet was prescribed for 8 h prior to PET investigation. The patients were positioned in a dedicated positioning device for the head with an additional cushion and bandages for fixation. A contrast media-enhanced (100 mL Ultravist 370 Schering) CT scan (detector collimation, 16 × 1.5 mm; tube current 100 mAs; tube voltage 120 kV; gantry rotation time 0.8 s) covering the entire head was performed for attenuation correction. PET was acquired in a single bed position with a 16 cm axial FOV with the middle of the FOV on the base of the skull. Emission scanning started 10 Min after intravenous administration of 200 MBq ^18^F-FET (acquisition time 20 min). PET emission data were reconstructed iteratively (OSEM algorithm) by using a 128 × 128 matrix.

 MR imaging of the skull was performed with the use of a head coil at a 1.5 T scanner (1.5 T Signa, General Electric, Milwaukee, USA, or 1.5 T Philips Gyroscan ACS NT, Philips, Best, The Netherlands). Regularly, magnetization-prepared rapid gradient echo (MP-RAGE) T1-weighted sequences after intravenous application of Gadolinium-DTPA (Magnevist, Schering AG, Berlin, Germany) at a dosage of 0.1 mmol/kg of body weight) were used for coregistration. This 3D volume dataset at a 1-(to 1.5-mm) slice thickness offers high spatial resolution and allows for coronal and sagittal reformations enabling contouring in orthogonal planes. PET data were obtained in 3-dimensional mode using a hybrid PET/CT system consisting of a multislice CT and a full-ring PET scanner (Biograph 16, Siemens Medical Solutions, Erlangen, Germany). PET/CT and MRI data were coregistered automatically using the treatment planning software BrainSCAN v.5.1 (BrainLAB AG, Feldkirchen, Germany) and a mutual information algorithm. Radiotherapy was usually administered at a fractionation of 5 × 2.0 Gray (Gy) until a dose of 60 Gy for the initial (large-field) treatment, followed by additional doses at a reduced (boost) volume, thereafter, in the range of 10 to 12 Gy at the reference point [13].

**Figure 1:**
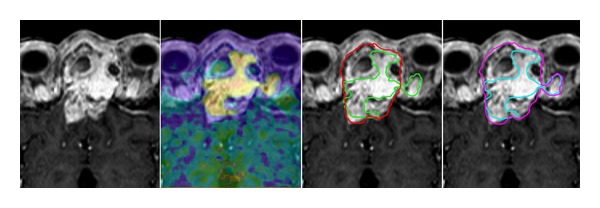
A 68-year-old male patient (patient no. 3) with a recurrent esthesioneuroblastoma and infiltration of the anterior skull base and frontal lobe. Parts of the GTV_MRI/CT_ showing equivocal hyperintensity represent cystoid scar formation, which is not enhanced in PET. MRI failed to detect the infiltration of the left orbit. The GTV_MRI/CT_ is delineated in red and the GTV_PET_  in green. The GTV_composite_ is lined in magenta and the reduced GTV_boost_ in turquoise. The depth of infiltration of the frontal lobe is demonstrated in equal size by both MRI and PET in this case.

 The retrospective segmentation and analysis of volumes were conducted according to the method published previously by our group [13], were complemented by definitions used by Grosu et al. [6], and are illustrated in Figure 1. We retrospectively performed delineation of the GTV on contrast enhanced T1-weighted MRI images of 14 patients previously treated with FSRT. We defined the GTV_MRI_ and expanded it by areas showing signs of erosion of adjacent bone in the CT component of PET/CT, leading to the composite volume GTV_MRI/CT_. Thereafter, the radiation oncologists were blinded to the generated contours. The volume GTV_PET_ was defined only in areas with FET-tracer enhancement. For delineation of GTV_PET_, we performed the same procedure as employed from Astner et al. [14], defining tumor borders by adapting the windowing to reach the alignement of PET and MRI in the tumor to normal brain interface. We formed the GTV_composite_ based on MRI/CT and enlarged it by the volume of PET not visible in the MRI/CT (GTV_PETplus_), which was justified because of the high specificity of FET tracer [5]. This GTV_composite_ was determined for the initial (larger) radiation fields. For these fields, we did not exclude nonenhancing areas with tumorous criteria by MRI due to the reported low sensitivity of FET [5]. We simulated the generation of the GTV_boost_ for the additional radiation dose, based on the GTV_PET_, assuming high tumour cell density [9] and/or high proliferative activity significant parts of the GTV_MRI/CT_, which showed hyperintensity but not enhancing tracer, were excluded from the high dose volume and were assumed to represent fibrosis, necrosis, or scaring after surgery and/or radiotherapy with reduced cell count and represented the GTV_MRI/CT minus_. 

 The data were evaluated on a lesional basis with the objective to compare the results of the conventional GTV (GTV_MRI/CT_) with the adjusted GTV_composite_, modified according to the PET information, and with the limited GTV_boost_. Changes to the conventional GTV or composite GTV > 10% were defined as significant and considered relevant for radiation planning. The statistical software R, version 2.11.1 (R Foundation for Statistical Computing, Vienna, Austria) was used for statistical analyses.

## 3. Results

**Figure 2:**
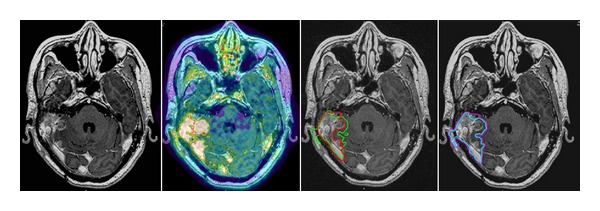
A 50-year-old male patient (patient no. 13) with a recurrent squamous cell cancer of the right petrous bone. The extent of infiltrative intracerebral extension was not delineable to the full extent by MRI. The MRI/CT volume is delineated in red and the PET volume in green. The additional PET information is included in the initial treatment fields (magenta). Note the reduction in the boost volume delineated in turquoise.

**Figure 3:**
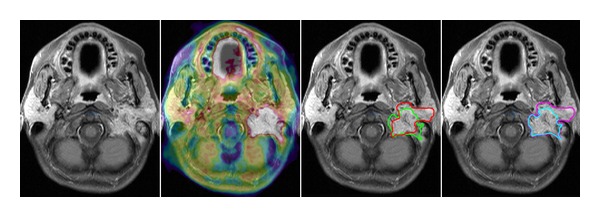
A 47-year-old female patient (patient no. 2) with a recurrent auditory canal cancer located at the base of the skull. FET-PET shows infiltration into the left parotideal gland to a lesser extent than assumed from the MRI. The MRI/CT volume is delineated in red and the PET volume in green. The additional PET information is included in the initial treatment fields (magenta). The boost volume could be reduced as delineated in turquoise.

 Visualisation of the tumour was possible in all CT (*n* = 14), and MRI scans (*n* = 11). FET tracer enhancement was found in tumours of all histological types in this study (Table 1). Infiltration of bone structures of the scull base was observed by conventional imaging and in the FET-PET in all patients; while, infiltration of the brain was observed in six patients, which was verified by both modalities. PET added target volume extension in terms of infiltrative growth into bone, soft tissue, or the brain in half of the patients (GTV_PETplus_) (Table 1). In three patients, there was clinically significant enlargement of the GTV from PET information (>10% of GTV_MRI/CT_). The mean GTV_PETplus_ accounted to about 1 ± 2 cm^3^adding about 5% (of conventional volume) to the GTV_composite_. FET-accumulating intracranial tumour parts with infiltration of the brain were demonstrated by FET-PET in 6/14 patients. In one patient, the true extent of infiltration of the brain was displayed only in PET (Figure 2). The restricted boost fields were based mainly on the GTV_PET_ volume. About 7 cm^3^ of the GTV_MRI/CT_, which showed no FET accumulation, could have been excluded from the high dose region (Figures 1 and 3). The resulting GTV_boost_ would have been on average smaller than the initial treatment field (GTV_composite_) by about 25%.

 To summarize our findings, the inclusion of FET-PET lead to significant (>10%) changes in the initial treatment fields in 3/14 patients and showed an additional tumour volume relevant for radiation planning. In 12/14 cases, FET-PET would have led to a subsequent decrease of more than 10% of the initial volumes for boost fields. The initial fields and boost fields remained unchanged in 11 patients and two patients, respectively.

## 4. Discussion

 When comparing our results with the available literature, an important problem that needs to be considered is the time interval of the PET scanning after FET injection. Malignant tumors, for example, glioblastomas, exhibit an early peak of FET uptake after 15–20 min which is followed by a decreasing time activity curve [15, 16]. In our study, the FET PET was acquired 10 to 30 minutes after injection of FET and our results can be compared to the studies by Grosu and Weber [6, 17], where the FET PET was acquired 20–40 min after tracer injection. These two studies were able to demonstrate enhancement of brain metastases with various histologies, confirming our findings of FET enhancement within histologically different SB tumors (Table 1). Therefore, some of the tumors in other studies may have been rated as negative in the late scans although the tumors might have been positive in the early scans. For example, in a study where scans were started one hour after injection of FET, Pauleit et al. [18] could not detect uptake of FET in the majority of extracranial tumours apart from squamous cell carcinomas.

 In extracranial tumors, to our knowledge, there are no studies comparing MRI and FET-PET/CT. Data are available for comparison of FET-PET with FDG-PET in patients with head and neck tumours. Balogova et al. [19] reported significantly greater sensitivity with FDG-PET and a significantly greater specificity with FET-PET. Pauleit et al. [5] confirmed the lower sensitivity of FET-PET (75% versus 93%) and reported a substantially higher specificity (95% versus 79%) in comparison to FDG-PET. In a similar approach, Haerle et al. [20] reported a sensitivity and specificity for FDG-PET of 89% and 50%, respectively, as opposed to 70% and 90% for FET-PET. Yet, the acquisition protocols of these three studies [5, 19, 20] were with late scanning of 60 min in each study, different to our method of early scanning, and thus decreasing the comparability to our results is limited.

 For visualizing the intracranial and intracerebral tumour extension of head and neck carcinomas, FDG-PET might, in comparison to FET-PET, have limited value if considering the high glucose metabolism of the brain. In a study conducted by Ng et al. [21] for nasopharyngeal carcinomas the results were discordant when comparing MRI with FDG-PET. There were findings of positive MRI (and negative FDG-PET) for infiltration of bony structures in 9% and 7% of the patients, respectively, and of negative MRI (with positive FDG-PET) for intracranial extension in 14% and 1% of the patients, respectively. The extension of brain metastases as depicted by FET-PET, in contrast to brain tumours, generally correlates with the extent as is visible by MRI. Yet, Grosu et al. [6], for differentiation of tumour from treatment related changes, found a possible specificity of FET-PET evaluated in a small group of four cerebral metastases. In our study, in one patient with recurrent squamous cell carcinoma of the petrous bone, the extent of brain infiltration was not consistently mapped by MRI and PET (Figure 3).

 For delineation of GTV_PET_, we performed the same procedure as employed from Astner et al. [14], defining tumor borders by adaption of the windowing to reach the alignment of PET and MRI in the tumor to normal brain interface. This method has been suspected to be subjective to a certain extent. Yet, as discussed by Bayne et al. [22], there are a lot of observations speaking against (semi)automated contouring by the use of cut-off values base on the maximal SUV or tumor- to background ration. The definition of percentages of SUV values proved difficult also in the work by Vees et al. [23] where in gliomas SUV cut-off based segmentation techniques performed poorly. We would agree to Bayne et al. in the assumption that an approach combining automated methods with visual contouring might be more reliable [22].

 There could be an additional impact of FET-PET to radiotherapy planning apart from the delineation of tumor extension. Biologic imaging into radiotherapy planning is included with rising frequency with the aim to adapt the dose distribution to tumour activity. It is assumed that high SUV values represent volumes with high cell density, and the contribution of selective dose escalating has been demonstrated in a study by Rickhey et al. [24]. For FET-PET, the correlation of SUV values and the cell density as has been demonstrated by Stockhammer et al. [7] and Derlon et al. [8], for MET-PET by Okita et al. [9].Yet, we are aware that until now, the relationship of FET uptake and cell density has been shown in gliomas but not in squamous cell carcinomas.

## 5. Conclusion

 In our study, the potential contribution of FET-PET/CT in the delineation of the GTV was assessed in 14 tumours involving the skull base. Due to its high specificity, FET-PET was able to add information about tumour extent that was not visible in conventional imaging. As to be expected, FET-PET added valuable details concerning infiltration of the brain. Using the correlation between enhancement and cell density, FET-PET provided useful information in a simulative approach to delineate the region of added dose. The comparison of the potentials of FET-PET and FDG-PET in the cranial base is the subject of another study in our institution FET-PET imaging proved to be a sensitive and specific tool in locating the active tumor burden, which may at least lead to a modified target volume definition to spare toxicity.

## Figures and Tables

**Table 1:** Patient and tumour characteristics and volumetric results of MRI/CT and FET-PET in 14 patients with skull base tumours.

Patient	Sex	Age	Histology	Diagnostic modalities	Location	Infiltration of bone	Infiltration of brain	Previous therapy	GTV_MRI/CT_ (cm^3^)	GTV_PET_ (cm^3^)	GTV_composite_ (cm^3^)	GTV_common_ (cm^3^)	GTV_PET plus_ (cm^3^/%)^1^	GTV_MRI/CT plus_	GTV_MRI/CT minus_ (cm^3^/%)^1^	GTV_boost_ (cm^3^)	GTV_boost_ (%)^2^	Bidirectional change of GTV_boost_ (cm^3^/%)^3^
											GTV_intial_^4^					GTV_Boost_^4^		
1	F	57	Adenoid cystic	CT MRI PET	Skull base	Petrous bone	None	Surg Rad Chemo	100	81	106	77	6/6	6	17/17*	89	84*	23/26*
2	M	47	SCC	CT MRI PET	Auditory canal	Posterior scull base	None	Surg Rad Chemo	104	62	105	50	0.5/0.5	17	37/36*	68	65*	38/56*
3	F	58	Esthesio-neuroblastoma	CT MRI PET	Anterior skull base	Anterior skull base	Frontal lobe	Surg Rad Chemo	84	80	84	64	0/0	1.5	19/23*	66	78*	19/29*
4	M	75	SCC	CT PET	Maxillary sinus	Maxillary sinus	None	None	127	130	127	99	0/0	11	18/14*	110	87*	18/16*
5	M	61	Chordoma	CT PET	Sella	Sphenoid bone	None	Surg	0.5	1.6	0.5	0.5	0/0	0	0/0	0.5	100	0/0
6	F	48	SCC	CT MRI PET	Naso-pharynx	Anterior skull base	None	Surg Rad	27	21	27	17	0/0	2	8/30*	19	70*	8/42*
7	F	79	SCC	CT PET	Sphenoid sinus	Sphenoid bone	None	Rad	11	13	11	4	0/0	1	6/55*	5	45*	6/120*
8	F	24	Sarcoma	CT MRI PET	Cranio-facial	Cranio-facial	None	Surg Rad	6	8	7	5	1/17*	0.5	0.5/8	7	93	1.5/23*
9	F	55	SCC	CT MRI PET	Naso-pharynx	Anterior skull base	None	Surg Rad Chemo	34	22	34	13	0.4/1	6	15/44*	19	56*	15/79*
10	M	47	SCC	CT MRI PET	Cavum nasi	Anterior skull base	Frontal lobe	Surg Rad Chemo	4	5	5	2	1/25*	1	0.4/10	4	80*	1.4/35*
11	F	53	SCC	CT MRI PET	Naso-pharynx	Anterior skull base	Frontal lobe	None	57	48	57	39	0/0	9	10/18*	48	84*	10/21
12	M	73	SCC	CT MRI PET	Naso-pharynx	Anterior skull base	Frontal lobe	None	26	21	27	16	1/4	1.5	8/31*	19	69*	9/49*
13	M	50	SCC	CT MRI PET	Petrous bone	Petrous bone	Cere-bellum	Rad	33	8	38	23	5/15*	3	7/21*	31	82*	12/39*
14	M	72	SCC	CT MRI PET	Skull base	Sphenoid bone	Temporal lobe	Rad	28	21	30	15	2/7	5	8/29*	22	73*	10/46*
Mean ±SD		58 ±15							45 ±42	37 ±38	47 ±42	30 ±31	1/5 ±2/±8	5 ±5	11/24* ±10/±15	36 ±35	77* ±14	12/41* ±10/±30

^1^% of GTV_MRI/CT_; ^2^% of GTV_composite_; ^3^% of GTV_boost_; ^4^Radiation field; *more than 10%. change from GTV_MRI/CT_.

F: female, M: male, SCC: squamous cell cancer; CT: computed tomography, MRI: magnetic resonance imaging, PET: positron emission tomography, Surg: surgery, Rad: radiation therapy, Chemo: chemotherapy, GTV: gross tumour volume, GTV_MRI/CT_: GTV by MRI or CT, GTV_PET_: GTV by PET, GTV_composite_: GTV by MRI/CT or PET, GTV_common_: GTV by MRI/CT and PET, GTV_PET plus_: GTV by PET not shown in GTV MRI/CT, GTV_MRI/CT plus_: GTV by MRI/CT not shown in GTV PET, GTV_boost_: GTV by GTV_PET_ and GTV_MRI/CT_, GTV_MRI/CT minus_: GTV only by MRI/CT excluded from final GTV, SD: standard deviation.
